# Book Reviews

**Published:** 1818-11

**Authors:** 


### CRITICAL ANALYSIS

### *OF RECENT PUBLICATIONS,*

IN THE

### DIFFERENT BRANCHES OF MEDICINE, SURGERY, &c.

*Transactions of the Association of the Fellows and Licentiates of the King's and Queen's College of Physicians in Ireland.* Vol. I. 8vo. pp. 494. Dublin.

*(Continued from p. 166.)*

### *Medical Report of the Fever Hospital in Cork-street, Dublin, for the Year* 1814. By John O'Brien, M.D.

WE have already entered into the consideration of the epidemic fevers of Dublin as fully as our limits will permit; we can, therefore, merely notice those observations, in this and the ensuing paper by Dr. Grattan, which may interest by their novelty or their particular practical importance.

Dr. O'Brien, as we observed in our Retrospect, commences with some remarks on the annual increase in the number of patients admitted into the hospital since its foundation, and gives some hints for a more effectual means of checking the progress of contagious fevers than can be effected by hospitals. With respect to the origin of the epidemic fevers, Dr. O'Brien thinks that the "simple and inflammatory bilious fevers" may arise from cold acting on depraved habits; but he is inclined to believe that the typhous fever is the peculiar product of contagion. In this opinion we by no means concur with Dr. O'Brien: we constantly observe the worst forms of what is called typhous fever appear under circumstances which disclaim a contagious source. Neither do we believe that there is any form of continued fever which may not have a sporadic origin; and for these opinions we would, if space allowed, produce the most ample authorities.

Dr. O'Brien is disposed to accede to the opinion which has more or less prevailed since the days of Hippocrates, that there are critical days on which fever terminates. In the more severe forms of fever, he says, he does not think that a crisis to recovery ever takes place before the eleventh or twelfth day. If it pass those days, we may look to the fourteenth as the next probable day; then the seventeenth and twentieth.

In evidence of the beneficial effects of blood-letting, it appears, since that practice has been more generally and freely adopted, although the number of patients admitted has increased, the mortality has considerably diminished. Dr. O'Brien, however, is disposed to restrict the use of general abstraction of blood to the first stage of fever. Blood taken from an artery, he says, has a much more debilitating effect than taken from a vein. This must be considered in a restricted sense: the loss of blood from an artery may perhaps be followed by a greater degree of immediate debility; it may more probably produce syncope; but its permanent debilitating effects are not greater than when it occurs from a vein. Purgatives, the author judiciously remarks, are useful in a two-fold point of view: by the power they have of regulating, in a certain degree, the particular determination of blood; and by keeping the bowels free from the vitiated secretions constantly poured into them during fever. Emetics were not often used, except when relapses were threatened; for preventing which, they were found particularly efficacious.

*Medical Report of the Fever Hospital in Cork-street, Dublin, for the Year* 1815. By RICHARD GRATTAN, M.D.

Dr. Grattan commences this Report with a description and history of the Fever Hospital, which are followed by some judicious remarks on the origin and character of the epidemic fevers of Dublin. He shews that, until a change be effected in the morals and general habits of the lower class of people, little can be expected from a hospital in diminishing the existence of fever.

The observations and opinions of the author have led him to consider the disease under four distinct forms. In the first, which is unaccompanied with local pain, and in which the stomach and chylopoïetic viscera are the principal seat of the disease, purgatives alone were found to be necessary. The kind most generally employed was a combination of calomel and ipecacuanha: the mineral acids were occasionally administered at the same time, without producing griping or any other unpleasant effects. Dr. Grattan is disposed to be very cautious in the use of blood-letting, particularly in pneumonia accompanied with typhous fever. "A single copious bleeding," he states, "has been productive of rapid effusion into the chest, or caused the fever to change from the inflammatory or bilious type to that of a putrid malignant form." We consider such consequences more likely to ensue from the want of having recourse to it in the early stages of the disease; and that a single copious blood-letting at that period is more beneficial than the abstraction of eight or ten ounces twice or thrice repeated, as advised by the author. To the forms of fever, accompanied with disorder of the chylopoïetic viscera, and with pneumonia, the author adds another, in which the sensorial functions are much disturbed, with watchfulness, delirium, low muttering, and stupor. In these cases, purgatives and blisters were the remedies on which the greatest reliance was placed.

Fever accompanied with local inflammation is made to constitute a fourth form of this disease. "These cases," the author states, "are fortunately those which are the least numerous; but still they often present themselves to our observation, and, though they sometimes afford conspicuous evidence of the value of our art, yet they too often give us cause to regret its uncertainty and inefficacy." The latter part of these remarks is too often verified in practice; but we think this may, in a great degree, be attributed to the principle which inculcates the possibility of the existence of fever, without local inflammation or congestion as its cause. Such an opinion necessarily induces a want of that careful investigation which is required to detect the existence and seat of local disease in epidemic fevers, particularly when affecting persons whose system has been much injured by intemperate habits. During the deranged state of the nervous system which accompanies this form of fever, a considerable degree of inflammation may exist, without affording the usual indications, and particularly when seated in a part on which pressure cannot be made: it is thence too frequently overlooked, until it has made a degree of progress that must necessarily prove fatal.

Dr. Grattan proposes a new classification of fevers: he would divide them into *synocha*, or simple inflammatory fever, without particular local disease; *synochitis*, fever with local inflammation; *typhus*, or low fever, accompanied with disturbance of the sensorium; and *typhitis*, the same, with local inflammation superadded. This arrangement we consider objectionable on many accounts; for, even acknowledging the possibility of the existence of inflammatory fever without particular local affection, it is evident that it would lead to a generalization of the mode of treatment, which must be productive of infinite mischief in practice. This form of disease has often, and not unaptly, been termed *Proteian*; the vigilance of an Ulysses should then be exerted to detect its changes, that it may be combated with security. It is not unusual to see *synochitis* become *typhitis* (to use the terms of Dr. Grattan) in a single day, from keeping the patient in a hot and impure atmosphere, the use of improper diet, &c. and *vice versa*.

We are not disposed to dispute about words when they do not tend to convey false ideas of what they are meant to signify; but, although many improper ones that "*jus et norma loquendi*" have established may be tolerated, yet such unmeaning terms as *synochitis*, and more particularly *typhitis*, should not be introduced into medical nomenclature.

We have differed in opinion from Dr. Grattan in some respects, and have nearly confined our remarks to those parts of his paper which we consider objectionable; it remains for us to inform our readers that it contains many valuable practical observations, and judicious and original views of the subject.

*Abstract of a Registry kept for some Years in the Lying-in Hospital of Dublin.* By JOSEPH CLARKE, M.D. Honorary Fellow of the Royal College of Physicians, and M.R.I.A.

We shall endeavour to deduce from this Report those facts which appear of most importance, and, by a general abstract of the result of different modes of practice in difficult cases, furnish some useful data for the regulation of practice.

On the subject of *ordinary* natural labours, Dr. Clarke says he has little to add to the excellent precepts clearly laid down, some years ago, by Mr. White of Manchester, and still later by Dr. Osborne of London. He is persuaded it contributes greatly to the safety of the mother and child to allow the uterus *gradually* to empty itself during delivery. Dr. Osborne, extending the doctrine of Mr. White, advises the expulsion of the body of the fœtus to be retarded by the accoucheur, in order to secure a more perfect contraction of the uterus. Dr. Clarke is disposed to adopt this mode of practice only when the uterus shews imperfect action in expelling the fœtus, in which case there is danger that the same imperfection may extend to the expulsion of the secundines, and then there can be no doubt of the propriety of the practice recommended by Dr. Osborne. Dr. Clarke has been in the habit, for some years, not only of retarding the expulsion of the fœtus in these cases, but, with a hand on the abdomen, of pursuing the fundus uteri in its contractions, until the fœtus be expelled. Such pressure also tends to prevent syncope. Labours conducted in this way are less liable to be followed by retention of the placenta, hæmorrhage, or after-pains, than when the birth is hastened or left to the efforts of the mother, frequently unnaturally excited by the exhortations of the attendants. In 10,387 cases, there were only twenty-one of retention of the placenta requiring manual extraction.

In *tedious labour,* unless it arise from the expelling powers being debilitated by disease, Dr. Clarke is decidedly averse to the use of the forceps; they were employed in only one in 728 cases in the hospital; and he says, it is so long since he has had occasion to use them in private practice, that he is persuaded a fair opportunity of doing it with advantage will not occur once in a thousand cases. When difficulty arises from mechanical causes, nothing but injury can attend the use of them.

When treating on *laborious parturition,* Dr. Clarke states three inches and a quarter from pubes to sacrum, to be the smallest diameter through which he had known a full-grown fœtus pass *entire:* in this instance the fœtus was putrid. When the pelvis measures less than three inches from pubes to sacrum, he is of opinion that it is good practice to perforate the head at an early period of the labour, and leave it some hours to be forced into the pelvis by pains, before the crotchet is applied. In 10,000 cases, only forty-nine ill-formed pelvises were observed.

In *preternatural labours,* he observes, that no instance of spontaneous evolution of the fœtus, as described by Denman, took place.

*Uterine hæmorrhage* is noticed in the registry to have occurred in fourteen cases before, and ten after, delivery. Of the former, four arose from a portion of the placenta presenting at the os uteri: one of these was a case of first pregnancy; one occurred in the sixth, and one in the eighth, month. In another there was deformed pelvis, consequently the head was perforated; the mother recovered. In the three first mentioned cases labour was forced, and one died (the patient in her first pregnancy). Of the remaining ten cases occurring before labour, four had delivery forced, one died; one had defective pelvis, the perforator was used, the mother died; one had a cross presentation, the fœtus was turned, the mother died; two had the membranes ruptured at an early stage of labour, both recovered; two were left entirely to nature, and one died. Not one case of hæmorrhage after delivery proved fatal.

*Convulsions.*—Of nineteen cases, fifteen happened to patients carrying single children, four to those bearing twins. Thirteen of the former took place before delivery; of those, six were delivered by the perforator and crotchet,—all recovered; two were delivered by forceps, one died; five were trusted to nature, and all recovered; one died in a fit after delivery. Of the four twin cases, one was delivered by the forceps, and died; three were left to the efforts of nature, one died. Sixteen were cases of first pregnancies.

Dr. Clarke observes, that, in looking over the cases on record, it appears that, when the operation of turning has been resorted to, the event has been less successful; and, therefore, when the mother's life appears in danger, and the forceps, or lever, cannot be safely applied, the perforator and crotchet seem to be indicated. Dr. C. has not much confidence in the power of medicine to relieve this affection. Bleeding in a few cases was found useful, by diminishing plethora; and in all it tends to prevent the bad effects of blood accumulated in the head by repeated paroxysms. He had not witnessed the slightest advantage from opium, except in checking the recurrence of the paroxysms after delivery. Acrid purgatives and glysters produced good effects, especially by their tendency to excite labour-pains.

Of twenty-one cases of retention of the placenta, twelve were of the first pregnancy,—not one happened with twins; five were accompanied with the hour-glass contraction of the uterus. Of four who died, three bore first children; seven were accompanied with flooding, from which one died. After waiting two hours, and using such gentle means as are commonly employed to promote the expulsion of the placenta without effect, little is to be expected from the efforts of nature. The patient has the best chance of recovery from a prudent interposition of art. When it is retained from want of contraction of the uterus, the object is to excite this by the stimulus of the hand; but, when it arises from contraction of the middle of the uterus, this should be gently distended; and, if much force be required, the effect of a large dose of opium should be waited for before it is persisted in. When it arises from morbid adhesion, Dr. Clarke says he is utterly unable to advise what should be done. This, fortunately, is a rare occurrence.

*Laceration and gangrene of the urethra and bladder*, from long pressure of the head on one part, occurred only five times in 10,000 cases; all were first pregnancies.

*Laceration of the vagina and uterus* happened eight times in the same number of cases. This usually took place at the anterior part of the vagina, near its junction with the uterus. One of the women recovered. In this case the fœtus, which had escaped into the cavity of the abdomen, was extracted by the feet through the ulcerated opening; which is the practice Dr. Clarke advises to be generally adopted.

*Prolapsus of the funis* proved fatal to the fœtus in three-fourths of the cases in which it occurred. The author has seldom found it practicable to afford assistance by any of the means usually recommended for re-placing the prolapsed cord.

We cannot dismiss the present work without expressing our hope that it will ere long be succeeded by others from the same source. Like all Transactions of Societies, it contains matter possessing various degrees of merit; but, taken as a whole, it is a highly valuable addition to the medical literature of the present period.

---

*Practical Observations on the Action of Morbid Sympathies, as included in the Pathology of certain Diseases: in a Series of Letters to his Son, on his leaving the University of Edinburgh, in the Year* 1809. By ANDREW WILSON, M.D. Kelso. pp. 406. Edinburgh.

*(Continued from page 250.)*

THE author now enters upon the consideration of such morbid sympathies as present themselves either in the course of certain particular diseases, or, in other cases, form the chief phenomena of the diseases themselves. Amongst the most obvious of these trains of connected symptoms, are those which associate themselves with affections of the chylopoïetic system. The great extent of sentient surface, through the tract of the alimentary canal, and its peculiar liability to be exposed to a great variety of irritants, make it little surprising that in this part of the system we should most frequently meet with the first deviations from healthy action. The extensive nervous intercourse established betwixt the rest of the system and the digestive organs, with the important rank these organs hold in the animal economy, prepare us to expect that a widely spread influence should emanate from them. This is the fact; and on this fact the author builds much of his reasoning, and of his consequent practice. In speaking of the reciprocal sympathies of distant organs, he observes—

"The morbid sympathies which subsist betwixt the stomach and certain distant parts are no doubt reciprocal; but it is observable, that the sympathetic affinities in these parts, flowing from irritation in the stomach, &c. are far stronger and more direct than those which affect the stomach from a distant origin. Upon the whole, however, the great object in practice is not so much to determine the specific form of the sympathetic affection, as to decide whether the morbid phenomenon presenting itself is really sympathetic, depending upon affinity, with irritation in some distant part; or whether it be idiopathic, and the effect of a primary local derangement in the organ, where the pain or uneasy sensations are seated; or whether it proceed from both of these causes acting together."

Dr. W. again calls the attention of his son to one of the leading phenomena of morbid sympathies, the affection, from consent with distant irritation, of membranous structures; this affection he affirms to be spasmodic; and precursive of the establishment in such structures of inflammatory action. This appears to be a favourite theory with the author; and, though we must confess our inability to conceive the *modus existendi* of spasm in serous or mucous membranes; yet, as we are not prepared to say what is precisely the deviation from a perfectly healthy condition in these structures, which marks the incipient stage of inflammation; and, as theory in this instance is not decidedly influential on practice, we may, perhaps without detriment, for the present allow this hypothesis to supply the interrupted link in a chain of dependant phenomena. Inflammation, when established, rouses the action of the arterial system, and symptomatic fever comes on.

"But spasm does not appear to operate as a direct cause of pyrexia of itself, till it has in the first place induced local inflammation. It is therefore probable, that the stricture on the skin, which accompanies feverish paroxysm, is no cause of fever, but merely a morbid sympathy depending on irritation, applied to some distant part of the body, most commonly within the intestinal canal; and, moreover, it is often observed to exist without a quickened circulation."

The arid surface which exists in the early stage of febrile action is, we conceive, one of the effects of the primary disturbing cause, by which all the actions of the system are thrown from their healthy balance, and a derangement of many of the most important functions is effected. Of this derangement none more early nor more obviously partake than the functions of secretion. The defective action of particular organs subserving this office may become, by withholding healthy or by imparting morbid stimulus, the generator of farther derangement; but such excitants are not to be considered as constituting or forming part of a primary morbid action. The suspension of exhalation from the capillary arteries of the skin will be productive doubtless of other disorder in the system; but the derangement of cutaneous function is itself but a consequence of preceding morbid excitement. We therefore coincide entirely with the author as to the probability of a dry skin not being a cause of fever; though we cannot so distinctly detect in it a proof that febrile action is not immediately consequent on spasm. We are not able to satisfy ourselves that spasm has been in this instance present; therefore, we cannot reason on its influence in the induction of fever.

The author's views of febrile diseases, as mainly influencing his practical deductions in the present volume, it will be right to give in his own words.

"You will remember then, that a febrile state, or a permanent increased action of the heart and arteries, may be derived either from irritation applied to the nerves of some distant part of the body, between which and the vascular system a primary affinity subsists, exciting that system into violent action by morbid sympathy; or from the effects of something of an irritating nature having been received into, and circulating with, the blood, and thereby directly applied to the internal surface of the heart and blood-vessels themselves. But the establishment of a febrile state of the system, as derived from remote causes within the body, appears very demonstrably to acknowledge the following primary distinctions.

"1st. As derived from the effects of acrid matter applied to some department of the nervous system, and exciting a morbid action of the heart and arteries by morbid sympathy; but such a remote cause, experience shews, is most commonly seated in the digestive organs.

"2d. As derived from the effects of irritation, arising from organic obstruction, somewhere existing in the body, and exciting increased action of the heart and arteries by morbid sympathy.

"3d. As derived from the effects of something of an irritating nature received into, and circulating with, the blood; being thereby directly applied to the internal surface of the heart and arteries.

"4th. As derived from passions of the mind, exciting an increased action of the vascular system, by morbid sympathy.

"5th. As derived from two or more of these primary causes of fever acting at the same time."

The first of these causes of febrile action, gastric irritation, which also calls into action other sympathies than that of the vascular system, is the one which the author is more immediately engaged in investigating. He proceeds to trace the progress of symptoms supervening on an obvious state of gastric disorder, and forming ultimately a decided paroxysm of fever; and then demonstrates the correctness of his pathology, by shewing the cessation of the connected morbid actions on the removal of irritating matter from the primæ viæ. To effect this is therefore the leading indication in gastric fever; and, for the purpose of procuring this salutary discharge of offensive matter from the first passages, the author says, we cannot safely trust to the operation of the more lenient emetics and cathartics, but must have repeated recourse to the action of the more powerful remedies of these classes.

"Accompanying the accession of fever, there appears to be established, in a greater or less degree, a spasmodic stricture on the excretory vessels of the skin, and, along with it, a defect of the natural quantity of perspiration; there happens, at the same time, either an increased evolution or a morbid retention of caloric in the system, perhaps partly of both, raising the general temperature of the body above its healthy standard."

And again, in another passage, he says,

"From the action of what cause it happens that caloric, when evolved, is also so tenaciously retained in the body under pyrexia, is not very clear; it is however evident, that, when sweating takes place, this excess of caloric flies off along with the matter of perspiration."

Without disputing with the author on the proximate cause of the suppression of the cutaneous excretion, it is certain that the suspension of this evacuation in fevers is followed by, and may justly be considered as, a principal cause of an elevation of the temperature of the whole body. Not looking to any set of organs in the animal economy for the calorific process; nor to any moving or conducting medium for the distribution of disengaged heat, it is palpable, that the aggregate actions and interchange of principles within the machine evolve continually a quantity of sensible caloric; for they maintain in the system a temperature elevated much above that of surrounding bodies. This heat-productive power in the system, to guard against a noxious and destructive accumulation, is balanced, in a healthy state, by the heat-absorbing processes of perspiration and evaporation from the surface. Arrest the secretion of the cutaneous fluid, and one of the natural guards against accumulated caloric is withdrawn, and a powerful stimulant is generated and diffused through the system. In the artificial supply of moisture to the skin by affusion, ablution, or immersion, we minister to a natural want, by occasioning the abstraction of superabundant caloric in the refrigerant process of evaporation. If the disengagement of heat in the animal body be commensurate with the actions going on, we might infer, that, during the excitement of a febrile state, an increased evolution of caloric would take place. But we are inclined to measure the quantity of caloric set free, not by the mechanical actions of the system, that is, by the velocity with which the fluids are propelled through the vascular apparatus; but by the quantity of chemical changes effected in the fluids, that is, by the extent of the secretions; and these in a febrile state are diminished. But, be this as it may, we have a sufficient cause of accumulation of heat in the system during fever, in the diminished abstraction of it by cutaneous evaporation.

But our limits will not permit us to follow the author too minutely in his reasonings, nor to dwell on all the points where we cannot entirely coincide with him in his hypotheses. It is very possible that experience may lead to an excellent course of practice, which may not be quite satisfactorily explained on theoretical reasoning. We shall be therefore obliged to pass over some hypotheses, which, we confess, do not come with conclusive force to our judgment; and confine ourselves to a cursory display of the author's leading views.

The diseases to which Dr. W. limits his attention in the present volume are all of an active character; in which the general moving powers of the constitution are roused, and febrile action is established in the system. In diseases of this class, he considers that derangement occurs, either primarily in the stomach and connected organs,—whence sympathetic morbid influences are propagated in remote organs, or through the nervous and vascular systems, and the whole of the functions are drawn into disorder,—or he traces, from an idiopathic topical affection, a morbid sympathy, reflected on the chylopoïetic system; which, as from a second focus, spreads disorder through the whole frame. He views the symptoms which display themselves in the progress of a disease as series of consentaneous actions, sympathetically called forth by affinity with some previously deranged organ. Thus disease or irritation in one organ will excite a sympathetic affection in another, perhaps remote one; which will superadd to the original disease, the symptoms of the secondarily affected viscus. This latter may go on to induce morbid action in parts sympathising with it, and thus disease, propagated from point to point, diffuses itself through every action of the system. The succession of morbid affections is not defined; nor is the nature of the sympathetic action uniform. These circumstances are modified by constitution, and by individual idiosyncrasies. Dr. Wilson's mode of viewing disease of the character before alluded to, is shewn in his brief display of the phenomena of small-pox; as brought forward in illustration of the concatenation of symptoms in febrile diseases.

"This statement is well explained by attending to what occurs in the phenomena exhibited under small-pox, especially observable in the inoculated small-pox, in which the time of receiving, and the progress of the infection, can be perfectly ascertained. Here the specific poison received into the body forms the primary cause of fever; the commencement of which fever is soon followed by a morbid sympathy of the abdominal viscera with the irritation formed in the vascular system, whereby an increased secretion into the stomach and intestines is produced; and the irritation excited by this accumulation within the canal very quickly gives the appearance of a re-action on the heart and arteries, contributing to augment their febrile action, and thereby forming a supervening cause of fever. From the same source also (gastric irritation), a severe head-ache arises, when, if the brain itself happens to be included in the morbid sympathy, a set of symptoms depending on that affection, including extensive disorders of the nervous system, necessarily take place. Again, if the membranes of any of the abdominal or thoracic viscera have become the seat of sympathetic influence from the irritation established within the canal, local inflammation is readily established, with symptoms peculiar to the organ so affected; and all these follow in regular climax from the primary exciting cause—variolous infection."

Guided by these views of the reciprocal dependance of morbid actions, the author goes into the consideration of various diseases of the class pyrexia. In all those which come before him, his attention appears to be primarily arrested by the production and accumulation of irritating matter in the tract of the alimentary tube; and the first indication, which dictates to his practice, is the evacuation of the contents of this canal. Dr. Hamilton has long since instructed the profession in the extensive influence of intestinal accumulations in the production and aggravation of disease; and has shewn the important consequence of an effectual and early removal of the offending load, by the liberal use of purgatives. Dr. Wilson has superadded to this valuable practice the almost as free use of emetics. In the cases which he here details, we find him commonly commencing his treatment by the exhibition of an antimonial emetic: and this he often repeats. A free dose of calomel with jalap, followed by tartrate of potass, given at intervals, as the course of the disorder indicates, completes, in most of his cases, the removal of disease. Facts are too strong for hypotheses; but, when more than one mean is had recourse to, and a desired end is obtained, it is not always possible to assign to its real cause the effect produced. We cannot say that, in the cases here brought forward, emetics were not always efficient in removing an excitant of morbid action; yet we must own that the author's arguments are not all quite conclusive in our minds of the necessity of having so general a recourse to this class of evacuants as he seems inclined to recommend. But this observation applies to the author's reasoning, not to his practice. The issue of his cases is satisfactory; there is therefore little ground to question the propriety of his treatment of them. It is observable in the practical part of his work, that Dr. Wilson has rarely recourse to any remedies in aid of those on which he mainly relies, with the exception of an opiate occasionally interposed. He makes no point of correcting, exciting, or moderating secretions: to clear out the vitiated contents of the stomach and intestines is his leading object; and, on the accomplishment of this, he places his chief reliance. In one or two instances a doubt suggested itself to us, whether the attainment of his grand aim might not have been somewhat retarded by an early exhibition of opium; but these things are difficultly decided on from the record of cases.

On the whole, though we meet in the work before us with some points of theory which we might perhaps be inclined to dispute a little with the author; yet, in his manner of considering disease, and in the general practical inferences which he deduces, we recognise the man of accurate observation and deep reflection. System has an optical illusion to its inventor, which casts over the varied hues of nature one common tinge; so that the judgments framed on it demand some allowance from those who have not viewed the objects through the same medium. Dr. Wilson has found an identity of character in the phenomena of diseases, which others perhaps, who had not looked at them from the same point, might not have suspected to exist. His work, however, assists us in arranging a complex assemblage of facts; and will, we are sure, contribute importantly to the simplification both of theory and practice, with those who are still bewildered in the maze of diagnostic pathology.

[In the former part of this critique, Number 235, page 248, lines 5 and 6 from bottom, crotchets should have been inserted before "*this*," and after "*organs*,"—the passage being parenthetical, and not a part of the quotation.]

---

*Observations, with Cases illustrative of the sedative and febrifuge Powers of Emetic Tartar.* By Wm. Balfour, M.D. 8vo. pp. 92. Edinb. 1817.

The perusal of the above work has afforded us no common degree of pleasure: we have received useful information from the facts therein related, and feel gratified that so valuable a remedy has engaged the attention of a physician so well able to demonstrate its importance, and whose authority will doubtless obtain for it the consideration it merits.

One of the greatest bars to improvement in the practice of medicine, the author observes, is the supine belief that nothing can be added to our knowledge of the qualities of those remedies which have been long in use. Hence it is that the greater number of those who are stimulated by the noble ambition of distinguishing themselves as the benefactors of mankind, direct their efforts to the discovery of *new* remedies, instead of instituting experiments with those whose medicinal powers are demonstrated.

It was observed by Bacon, that knowledge more quickly springs from absolute ignorance than from error; so we are better acquainted with the qualities of several substances but recently employed as medicines, than with those which have been familiar to every practitioner from time immemorial. Dr. Balfour justly remarks, that the most skilful practitioners prescribe the fewest medicines; and that it is very probable, were the powers of those we already possess fully understood, that the tenth part of them would be more than sufficient for the purposes of practice. Physicians would then be like dexterous surgeons, who depend more on their own management and skill than on number of instruments.

Emetic Tartar, the author observes, has been in use ever since the introduction of chemical medicines into practice, and every author has been loud in its praise. It is very questionable, however, that its employment has been as universal as the encomiums on it would indicate. Several of the most eminent practitioners in Edinburgh, whom he has been in the habit of meeting for many years, never once hinted at the employment of antimonials, in even the most acute inflammatory diseases. On conversing with his medical friends on the subject, those who were in the habit of using it acknowledged that, though they knew Emetic Tartar to be an excellent medicine, in *nauseating doses*, as promoting perspiration without heating the system, yet they never entertained the view he has given of it. "The sedative and febrifuge powers of Emetic Tartar" was a phrase quite new to them.

"With Dr. Cullen, (the author continues,) Emetic Tartar was a favourite remedy. This appears from his First Lines. I have been told, but I do not believe it, that he carried this medicine an unjustifiable length. The only subject of regret, however, is his recommending it, wherever he speaks of it, in nauseating doses. Had not this been the case, the authority of so great a man must infallibly have rendered the employment of it much more general than it has yet been. Nausea is an unpleasant feeling; nor is it easy to induce patients to swallow the medicine a second time, which is sure to produce it. Practitioners, too, who suffer themselves to be influenced chiefly by appearances, would naturally conclude against a remedy which, unless exhibited with judgment and caution, is violent in its operation, and which, in the days of Dr. Cullen, was comparatively new. Hence, together with the aversion manifested to it by a great living character, the almost total neglect Emetic Tartar has experienced in this country. Even at this day, it is in nauseating doses only the remedy is recommended, and in constitutional derangement alone; or where local affection is so considerable as to give reason to apprehend constitutional derangement. I trust, however, to be able to show that Emetic Tartar is eminently efficacious in chronic, as well as in acute, disorders; in topical affections, as well as in general derangement; and that its efficacy, in either case, is not confined to *nauseating doses*. Nay, more, I will demonstrate that, in many cases of local inflammation, accompanied with violent re-action, blood-letting to one-third of the extent generally practised, is not necessary to the cure; that a speedy and perfect cure can be obtained, with the loss of so moderate a quantity of blood as to warrant the conclusion that it might be safely omitted altogether, even in circumstances in which it is generally considered the only means of saving the patient."

The beneficial effects of blood-letting in many diseases, Dr. Balfour remarks, depend more on the relaxation of the system induced, than the quantity of fluid abstracted: if, then, this effect can be produced by other means, will not a mighty object be gained? He then observes,

"If such a thing is possible, why should practitioners continue to abstract sixty, a hundred, two hundred, ounces of blood from a person labouring under pneumonia? A recovery after such practice is, in many instances, an escape, not a cure! Is it consistent with any principle that life should be reduced to the last ebb, without regard to age, habit, or constitution, on account of the recurrence of pain in the chest and difficulty of breathing, when we possess the means of equalizing the circulation, without producing corresponding debility? The Diathesis Phlogistica of authors, or the preternaturally-increased tone or contractibility of the arterial system,—sometimes induced by any considerable local inflammation, at others the effect of general causes, and always tending to congestion,—is most readily and effectually taken off by the relaxing power of blood-letting; which ought, therefore, in pressing circumstances, to be first employed. What I contend for is, there is no necessity, in even the most urgent case, for carrying the lancet the length of "giving the patient the chance;" that is, of nearly bleeding him to death in order to save his life."

Dr. Balfour, supposing that, in inflammatory diseases, the nervous system is primarily effected, of which the arterial excitement is the consequence, considers that the attempt to remove this without the aid of those remedies which more directly affect the nervous system, is not the most scientific mode of proceeding. "When re-action has taken place before the physician is called in, blood-letting may certainly be necessary, because increased arterial action, at first an effect, now acts as a cause; but certainly attention is even then due to the primary affection, and, if due attention is paid to it, its effects will be more easily controlled. The advocates for the unlimited use of the lancet, however, follow a more summary method. They cure their patients in the same way the Romans gave peace to the countries they invaded; and that was, by making the blood of the inhabitants to flow till they became perfectly passive.

Several cases are then related of the different diseases in which the powerful effects of this remedy have been evinced; of which we shall transcribe some of the most important, referring our readers to the work itself for more detailed information in this respect, and for the original and judicious reflections of the author on the diseases to which it is applicable, and its mode of action.

"Case 1.—Mr. S. aged 30, was attacked, on the 7th October, 1817, with pneumonia, for which he was bled to sixty ounces in three successive days; and, on the 15th, again to twenty ounces. Having got out in about three weeks after this, he was again seized with pain in the breast and difficulty of breathing, accompanied with much higher fever than in the first attack. He was bled to twenty-six ounces, with relief at the time; but, in twenty-four hours after, all the symptoms returned with increased violence. Afraid of the patient's strength failing, as his feet were now become œdematous, I was unwilling to carry blood-letting any farther. I therefore ordered an ounce of a solution of two grains of Emetic Tartar in six ounces of water, to be given every hour. The third dose produced sickness, and with it relief from pain. In four days the patient was free from complaint in his chest. About the middle of February he had another attack of pneumonia, which again yielded to Emetic Tartar, without blood-letting being premised. Had I, in this instance, continued to trust to the lancet for subduing the inflammatory action that existed, thoracic effusion would have been induced, and I would infallibly have killed my patient.

"Case 3.—Mrs. B. aged 55, a thin, delicate, complaining woman, was attacked in January last with pain in the chest, difficulty of breathing, and fever. She had struggled with her complaints some days before I was called. I found her extremely weak, pain in the breast fixed, severe, and impeding respiration to a distressing degree; pulse 100, small and hard. She complained also of being drenched in sweat every night, but especially towards morning, and on the head and superior parts of the body chiefly. I ordered her a solution of four grains of Emetic Tartar in eight ounces of water. Of this she was to take a tablespoonful four times in the twenty-four hours, unless such quantity sickened her; in which case the dose was to be diminished, so as not to occasion nausea. In five days the pain in the breast was removed, and she could make a full inspiration. Debility and night-sweats were now her only complaints. For these I had recourse to nitrate of silver in the form of pill,—a fourth of a grain a dose, three times a-day. The power of this medicine, in checking the sweating, was almost immediately felt, and the patient gained strength daily. In ten days from the time I was called in, I took leave of this lady, restored to a greater degree of health and strength than she had for a long time enjoyed.

"Case 8.—Mr. W. was attacked, on the 17th December, 1817, early in the morning, with pain in the great toe, at the instep, reaching through to the sole, and round the outer ankle of the right foot. I was called to him in the course of the day, and found the parts swelled, excrutiatingly pained, and of a fiery red. Pulse 80. From my patient having had several attacks of gout before, there was no room to doubt that his present complaint was of the same nature. I proposed to bathe the parts with spirits and water of a temperature with that of the parts; a practice I followed, particularly in the case of Sir Thomas Troubridge in 1815, before Dr. Scudamore published on gout. To this the patient objected, on the score of his having been treated in the same way once before, when he had a slow recovery. I applied compression with my hand, dipped in flour, for a few minutes, which he bore very well, though friction would have made him mad. Ordered a solution of two grains of Emetic Tartar in six ounces of water, of which he was directed to take an ounce every two hours.

"Dec. 18.—Passed a very restless night. Pulse 96, and hard, with stitch in the right side, increased by coughing or a full inspiration. Had taken very little of his medicine. I now informed him that, if he did not take his medicine as prescribed, I would be under the necessity of bleeding him freely, by which he might lay his account with being much longer confined than he otherwise would be. This had the desired effect, as he regretted absence from business much. This day he took his medicine steadily, with the addition of two drachms of compound powder of jalap. In the evening I found his pulse much fuller and softer, with an agreeable diaphoresis all over the body; stitch in the side declining, and the purgative had operated briskly.—Dec. 19th. Passed the night very well; no uneasiness from the foot; redness and swelling declining; pulse 86, full and soft. Continue the antimonial mixture in quantity to maintain a softness of the skin.—Dec. 20th. Pulse natural; stitch gone; bowels regular. But the patient cannot point his foot to the ground. Applied percussion gently all over the sole, and then a bandage. In the evening could walk a little. Repeated the operation and bandage. Next day, 21st, could walk pretty well. On the 22d, walked perfectly well.

"On the 25th, my patient sent for me to his counting-room, when he told me his right foot was as bad as his left had been, and that he could not point it to the ground, were he to be made proprietor of Edinburgh for so doing. Admitted he had got a fresh cold; pulse rising and hard. Applied percussion to the sole of the foot for some minutes, when the patient was immediately enabled to walk. Gave him a slightly-nauseating dose of his antimonial medicine. He dined in his counting-room, and walked home at eight o'clock. Passed an uncomfortable night. Next day resumed his antimonial medicine, none of which he had taken during the night. This day he took six drachms of sulphas. magnes. also.—27th. Was free from complaint, with the exception of being slightly lame. Two more doses, therefore, of percussion, exhibited on the morning and evening of this day, completed the cure; and the patient went abroad on the 28th in perfect good health, which he has enjoyed uninterruptedly ever since.

"Case 10.—Captain B. applied to me, on the 12th December last, for a rheumatic affection in his right shoulder, and left elbow-joint. He could neither put on nor off his coat without aid; and was deprived of sleep by the pain in the elbow attacking him, in the night, in paroxysms of such severity as to make him cry out. In ten minutes I gave freedom of motion in the shoulder; and the pain in the elbow was coerced in a considerable degree by a bandage, but not removed. On the 13th he did not go abroad, and when I visited him at 3 P.M. found a slight degree of fever present. Two grains of Emetic Tartar in six ounces of water were ordered; of this the patient was directed to take a table spoonful every hour, till nausea supervened.—14th. Had taken most of his medicine, without nausea or any sensible perspiration. Slept well, having had but one attack of pain in the night, which was instantly checked by percussion.—15th, 16th. Sleeps without interruption, and is free from complaint.

"Case 11.—About the middle of January, William Paterson, a poor lad, aged 19, came to my house at nine o'clock at night, with one arm of a ragged coat on and another off, complaining most grievously of a pain in his elbow-joint. There was much swelling round the joint, and a considerable way both above and below it. I handled the parts as the patient could suffer me, but had no bandage to apply. In a few minutes he began crying like a child. I asked if I had hurt him? He assured me not; but that he was sure a snow-shower was falling, for the pain was always much exasperated by such an occurrence. I looked out, and found his conjecture correct. I gave him three ounces of antimonial mixture, in which was a grain and a half of Emetic Tartar; directing the one half to be taken as soon as the patient got home, and the other half in an hour after. Next morning it was reported to me, he slept all night, a few minutes only excepted, when he experienced a slight paroxysm; and I ordered the medicine to be continued. On the third day after his applying to me I visited the patient, as the physician, whose care he was under before he came to me, had not, he said, called on him for some time. I found him lying at ease; the swelling reduced two-thirds; the pain, even to the touch, entirely gone, except in a single point in the bend of the arm. Six grains of Emetic Tartar, in twelve ounces of water, was the amount of the medicine taken. It produced not the slightest nausea, nor any observable increase of perspiration, for thirty-six hours. It then began to operate powerfully on the skin; but, before this took place, pain was completely subdued. Indeed, he had but one return of pain, and for a few minutes only, after he began the medicine. I applied compression to the pained point, first with my hand, and then with a bandage,—including the whole of the parts that had been swelled. Two more operations gave complete motion to the joint, and the patient was at his work in a week.

"Case 14.—A gentleman contracted gonorrhœa, in which the inflammation run pretty high, and the discharge was copious. Circumstances rendering it necessary to keep up appearances, he could not confine himself. I advised abstemiousness at table, ease, cleanliness, and a saline aperient occasionally. In a few days he got hernia humoralis. The affection had reached the scrotum, which had become red by the time the circumstance was communicated to me. I ordered suspension, and a solution of Emetic Tartar in water, of the strength of half a grain to the ounce. Of this mixture he took an ounce every two or three hours, and the progress of the complaint was immediately arrested. The cure, indeed, of the hernia humoralis was completed in one day. The medicine was continued in small quantity for some time, but not more than six grains of Emetic Tartar were taken, and there was no return of the complaint.

"Case 15.—A young gentleman, having contracted gonorrhœa, was greatly alarmed lest the circumstance should come to the knowledge of his friends; and therefore insisted on having an injection, that the cure might be the more speedy. In a few days the discharge had nearly ceased, when the patient was seized with pain and swelling in one of his testes. I prescribed Emetic Tartar in alterative doses, as the patient could not be confined. The pain soon abated, but was not altogether removed, on account of the extreme caution observed in taking the medicine, lest sickness should be induced by it. I remonstrated with him on account of his timidity, and he increased the dose till slight nausea was only once produced. He abandoned the medicine, and I abandoned my patient. He then promised compliance, resumed the medicine for a few days, and the cure was completed.

"Case 21.—"On the 2d of April, a young gentleman consulted me for a constant uneasiness he felt about the region of the bladder, and which was increased by the accumulation of water. He made water with difficulty and increased pain. Dated his complaint from exposure, some time before, to cold and wet. I was satisfied, from the *nisus* requisite in expelling the urine, that the neck of the bladder was principally affected. I put him upon Emetic Tartar, with little or no effect for some days. At length the medicine began to operate; not, however, by any other sensible effect than freedom from pain and facility of making water.

Two dozen of pills, each containing a fourth of a grain of Emetic Tartar, effected a complete cure."

The author concludes with observing, "From the facts narrated, it may be fairly inferred, I think, that Emetic Tartar must be highly beneficial in every genus and species of inflammation, whether chronic or acute; those affections excepted, in which the stomach is generally so irritable that it is not probable the medicine could be retained in sufficient quantity to have much effect on the circulation. We have seen its effects in symptomatic fever, induced by local injury,—in several severe cases of pneumonia,—in inflammatory gout,—in rheumatism, chronic and acute,—in cynanche tonsillaris,—in idiopathic fever,—in hernia humoralis,—in chronic inflammation of the bladder,—in inflammation of the mamma,—in ophthalmia,—in chronic hepatitis,—in nephritis: it certainly is not carrying analogy too far to anticipate similar beneficial effects from it in other kinds of inflammation, and in inflammatory affections of other organs."

In a private communication we have been favoured with from Dr. Balfour, he states that, from experiments he has already made, and is now prosecuting, he is confident that Emetic Tartar, judiciously exhibited, will prevent scrofulous inflammation and ulceration of the lymphatic glands; he therefore concludes, that it will prove not only a cure, but a preventive, of pulmonary consumption.

*Remarks on Burns and Scalds, chiefly in reference to the Principle of Treatment at the time of their Infliction. Suggested by a Perusal of the last Edition of an Essay on Burns, by Edward Kentish, M.D.* By Nodes Dickinson, of the Royal College of Surgeons; Staff-Surgeon to H. M. Forces; Member of the Medical and Chirurgical Society, &c. 8vo. pp. 134. London, 1818.

There is no species of external injury for which established principles for the treatment (and such principles as may be intelligible, not only to medical practitioners, but to the public in general,) are more to be desired, than that which forms the subject of the present work. It must appear extraordinary to those unacquainted with the difficulty of acquiring pathological knowledge, that an injury, the cause of which is ascertained, and the effects evident to the senses, should be so imperfectly understood. There is, indeed, none, however obscure, respecting which a greater diversity of opinion prevails.

Arguments, each persuasive when partially considered, have been advanced in favour of two opposite modes of treatment, the consequences of which have been productive of considerable mischief in practice. Many practitioners of that class on the conduct of which the welfare of general society principally depends, averse to reflection, or influenced by each of those series of arguments, take a middle course, and employ measures, in cases of extreme danger, which are at best but nugatory. An endeavour, therefore, to decide this important question must meet with no common degree of attention from all those who are anxious for the improvement of surgery.

It will, undoubtedly, be supposed by many persons, that this object has been effected by the Treatise of Dr. Kentish, considering that his theory is founded on actual facts, and his mode of practice attended with results, the superiority of which is admitted by the greater number of surgeons: this expectation has, however, not been fulfilled. The empirical use of remedies is not willingly had recourse to at the present period; their mode of action, and the principle of their application, are more or less enquired into by all practitioners. The theory of Dr. Kentish, unintelligible to many, visionary and otherwise objectionable to others, has met with numerous opponents: his mode of practice has also been censured by men of considerable talents; and it is with the view of establishing the objections to it, that Mr. Dickinson appears before the public on the present occasion. We, however, transcribe the preface, that the intentions of the author may be more perfectly understood.

"It is hoped the following pages may be useful to the *general* reader. The object is to compare those means employed in the treatment of Burns and Scalds that have occasionally been deemed either useless or hurtful, with others regarded more beneficial, or at least innoxious.

"From such a view, the number of valuable applications will be enlarged, by evincing the safety of such as are by some considered dangerous, and by affording an estimate of their relative virtues on the ground of experience, which has demonstrated their utility, and on the basis of science, which refers their curative power to the influence of a common principle.

"Many facilities may thus, it is hoped, be afforded towards alleviating the pain produced by injuries from fire at the moment of their infliction, when extra-professional aid must occasionally be resorted to in the absence of surgical skill.

"To the *inexperienced* of the profession, these remarks, it is likewise hoped, may be useful in the early hour of their research, and serve, at least, as a central point round which an increasing series of examples and reasonings may be hereafter drawn; completing the deficiencies, and correcting the errors, of what is now submitted to their review.

"To those whom experience has already enlightened, this small Tract is most respectfully presented. It is not addressed to the masters of the profession with the presumption of contributing to their knowledge, but in the trust that the relation of the facts and opinions it contains may yet deserve the sanction of their approval."

Mr. Dickinson commences with some reflections on the various modes of treating the injurious effects of heat, which have been employed in different countries at different periods; and endeavours to prove that the same final intentions have directed them all,—to subtract preternatural heat, and diminish excessive action. He then proceeds to the consideration of the theory and practice of Dr. Kentish. His explanation of the effects of cold on the body are first noticed: Mr. Dickinson very justly remarks, that Dr. Kentish's explanation of the cause of the separation of frost-bitten parts from the rest of the body, as expressed in the preface to the first part of the work, is by no means satisfactory. Dr. K. says, "When parts of the body, by the disease termed frost-bitten, have ceased to act in unison with the body to which they belong, great nicety is required to restore action to those torpid parts. If the general circulation of the body is increased before the circulation of the torpid parts is restored, a solution of continuity would be the consequence." p. xxiv. This Mr. Dickinson would explain in the manner Dr. Kentish has himself done in the fifth chapter of his work, in which, after having pointed out the mode of treatment, he says, "If these precautions are not attended to, and the stimulus of too much heat be suddenly applied, the parts will be excited to excessive action, and soon exhaust their vital powers; the consequence must then, of necessity, be a speedy mortification." p. 96.

The author then expresses his opinion of the nature of the injuries from the application of an undue degree of heat to the body, and the measures that should be adopted for their relief. He decidedly favours the use of general antiphlogistic means, and the topical application of cold or refrigerants. This mode of treatment necessarily arises from the view he has taken of this disease: the immediate effect of heat, to a certain extent, is considered to be inflammation; and the observations of various authors are adduced, who have maintained the same opinion. We shall quote the words of the author on this point, and his general remarks on the theory of Dr. Kentish.

"The opinion expressed, by the authorities quoted, of the *inflammatory* character of the injury produced by burns and scalds excited within the degree 'where the action of the parts is alone increased,' is strictly in unison with the experience which has led me to regard the local affection in that point of view, and the general derangement of the same kind.

"But Dr. Kentish expresses a different opinion, and builds his practice upon the consideration of a 'disparity of action between the injured part and the rest of the system.' 'The mode of relief (he observes) will therefore be to restore the unity of action, by gradually diminishing the increased action of the part, and by increasing the general action of the system.'

"In these cases of injury, 'caused by a pernicious quantity of caloric suddenly applied inducing increased action, the internal means of relief will be to administer those substances which will, in the quickest and speediest manner possible, throw the heart and arteries into the *most violent action compatible with life.* By these direct stimulants internally, the circulation will be carried to the greatest possible degree of quickness; by which means there will be infinitely less disparity of action between the part excited by the burn and the general system, than by any other manner of treatment.'

"Now, if the circulation be carried to *the greatest possible degree of quickness compatible with life*, inflammation must be the result. Dr. Kentish assumes that the cause of the mischief in burns arises from the system remaining in its ordinary degree of action, while the action of the part burnt is extraordinarily increased; disparity being thus produced between the action of the part and the whole.

"What proof is there that such disparity exists? and, if it does, why is such a disparity injurious? It is so erroneous to suppose that the imaginary destruction of this equilibrium produces the ill effects spoken of, that in reality they are produced by the very unison which is instantly established between the action of the system and of the injured part, occasioned by the immediate sympathising of the constitution. When the injury produces increased action of the part, if the example be one of much severity, the general system will sympathise; the action of the heart will be augmented, and synocha will result.

"If the injury be still more violent, the irritation will be so great as to exhaust the powers of the system generally, and mortification of the part will very probably ensue. Should the morbid excitement be subdued in the first example by antiphlogistic remedies, the health returns; so likewise in the second example, if cordials and stimulants are employed with a moderation proportioned to the measure of exhaustion. But, if in the first case violent stimulants are resorted to, visceral inflammation will most probably result; and, if the same violent stimulants are made use of in the second case to overcome exhaustion, they will either rapidly sink the remaining powers of the system beyond repair, or occasion a dangerous re-action.

"Dr. Kentish remarks, in opposition to the 'uniformity of opinions among all preceding authors:' 'As it appears to me that the too general acceptation of the similarity of burn and inflammation has led to erroneous conclusions, I must, therefore, venture to deny that a burn is an inflammation, though it may, in its consequences, produce an inflammation. I may be wrong in this assertion; but, at least, it has the merit of novelty for its recommendation.'—'I regard the first action of caloric upon the system as an irritation.'"

We perfectly concur with Dr. Kentish in the opinion last mentioned, and it is from the consideration of the disease in this point of view that we would endeavour to explain the superior efficacy of the stimulating mode of treatment.

The theory of Dr. Kentish, as expressed in his work, is, we confess, neither clearly intelligible nor satisfactory to us. We cannot see the necessity of raising the actions of the general system to such a degree, as to *equal* those of the injured part; nor do we consider that the danger in these cases arises from the temporary disparity of action. That hot oil of turpentine can *absolutely* diminish the action of a part, although a more powerful stimulus may have been previously applied, does not appear probable; it must continue to act as an *absolute* stimulus while the parts are susceptible of its power. The actions of a part may subside under its use, because, after a certain length of time, it will become less and less susceptible of its influence. It is probable, however, that our ideas on this subject accord with those of Dr. Kentish, and that this apparent disagreement arises from his having expressed his opinions in words not perfectly intelligible, even to those who are accustomed to seek for the *ideas* of an author, rather than attend closely to his *verbal* illustrations of them.

We agree with Dr. Kentish in the opinion, that the immediate effect of the agent in question is not inflammation: there is increased action of the parts, when the injury has only been to a certain extent, which increased action can only be supported by a proportionate supply of the principle of irritability; therefore, to diminish that, would be to remove the means of supporting the disease. The primary cause is, no doubt, the painful impression on the nerves; those means, then, which will relieve pain, must be considered the most advantageous. Here we must refer to experience, which we do not hesitate to assert has furnished facts in favour of stimulants, that are sufficient to decide the question. There is, however, something more to be effected:

the application of a stimulus causes a preternatural afflux of blood to a part, which is always accompanied with increased sensibility, and, it is reasonable to conclude, with increased nervous influence; this is attended with augmented irritability, and, consequently, action. When effects have existed a certain length of time, they will continue independently of their *original* cause, and produce a secondary series of effects, which often constitute the most strongly-marked traits of a disease: in these cases, to remove the primary effects, will be to remove the disease. The only question then is, in the case of burns, whether it is better to lower action by the abstraction of accustomed stimuli,—as the diminution of the usual heat of the surrounding medium and the quantity of blood, (for the only sedatives we are acquainted with are *relative*;) or to favour the expenditure of the irritable principle as fast as it is accumulated. We must here again refer to facts; which prove that abstraction of stimuli, to the greatest extent consistent with vitality, will not permanently depress action; it will at length ensue, from the power of the accumulated irritability, and will generally be followed by actual inflammation. On the contrary, when the irritability is expended from the effect of a stimulus, the parts become gradually less sensible to its influence, action decreases, and they return to the natural state. If the use of stimuli be continued too long, there will be a determination from the whole system to the part, both of blood and nervous influence, from which the irritable principle will be accumulated in a greater degree than can be expended by healthy action, and inflammation, or some diseased action nearly resembling it, will be the consequence. This, it may be observed, Dr. Kentish is very cautious to guard against, by directing that stimulant applications should not, in general, be continued longer than from twenty-four to forty-eight hours. We consider that the effect of the inward remedies advised by Dr. Kentish, may be explained on the same principle:—an increase of expenditure of irritability by the general system is produced, and, consequently, accumulation of it in any particular part is prevented.

We have argued thus far theoretically, or rather hypothetically, on the subject, because we consider that such a view of it satisfactorily explains the mode of action of stimulants, and their beneficial effects.

The propriety, however, of those measures does not require the support of arguments,—it is decidedly evinced by experience, when employed in the manner, and under the regulations, so judiciously advised by Dr. Kentish. The ill consequences which have been attributed to his mode of practice have arisen from its being employed in particular cases to which it is not applicable,—as when inflammation had taken place. There is not a clearer evidence of the beneficial effects of any mode of treatment, than that its relieving pain; and, that this is most effectually done by stimulants, every day's experience will prove. Stimulants frequently produce an increase of pain on their first application, but this only continues for a short period: the use of cold is productive of temporary ease, but it is followed by an increase of pain, which at length, when the injury is severe, cannot be subdued by the most assiduous and frequently-repeated application of that agent.

The stimulating plan of treatment is almost universally had recourse to in the collieries in the north of England, which would not be the case were there not the most decided evidence of its superior efficacy. It is the general practice in glass-houses, where burns constantly happen, to hold the part to the fire until the pain is relieved: this practice is efficacious in all cases where the injury is only to a certain extent. In those instances where, from the shock given to the nervous system at the time of the accident, no re-action takes place, but the patient lies in a state of stupor, the stimulating plan is the only one that can be productive of benefit.

We have, in some measure, digressed from the direct consideration of the work before us, but we hope not uselessly; certainly not so, if the observations we have made excite reflection in the minds of our readers on this interesting and important subject.

On resuming the work of Mr. Dickinson, we feel it necessary to remark, that he has treated the subject in a manner which does honour to his acquirements, liberality, and candour; and, acknowledging his principles, it must be allowed, with much soundness of judgment. He evinces a perfect acquaintance with the works of the best writers, and makes several original reflections, and useful practical remarks, that are worthy of attention even by those who may not concur with him in all his opinions. To give a regular analysis of this work would occupy a space beyond what our limits will admit, as almost every page contains remarks on some points of the theory and practice of Dr. Kentish, which would lead to endless discussion; we shall, therefore, merely transcribe the general conclusions of the author, in his own words, referring our readers to the work itself for his particular arguments on this subject.

" *Conclusion.*—1. The terebinthinate dressing to burns and scalds does not appear to be either new or peculiar in its remedial action, or so particularly beneficial as to recommend its adoption to the exclusion of many other applications.

" 2. The details of Dr. Kentish do not appear unfavourable to the employment of refrigerant applications; those of many other writers, and my personal experience, establish their utility, as well as perfect safety.

" 3. The evidence adduced to point the pernicious effects of the antiphlogistic treatment, I think, must be deemed inconclusive; while the necessity for its employment on certain occasions, I trust, has been made sufficiently apparent by the various testimonies advanced in the preceding pages.

" 4. The internal stimulant practice, when employed to the full extent enjoined by the theory of Dr. Kentish, appears to have failed.

" 5. When the stimulant treatment succeeded, it does not seem to have been carried to so great an extent. It does not appear to have been adopted except in cases of severity, in which, from the detail of symptoms, we must consider a great degree of exhaustion to have been produced by the violence of the injury. In which case moderate stimulation was indicated; but, upon a principle that would make its application pernicious in those numerous examples of less severity, in which there merely ensues inflammation, —'only increased action.'

" 6. This view of the subject affords great satisfaction; inasmuch as it provides for a ready assent to the accuracy of the practical records adduced in the Essay on Burns, although the theory proposed should be deemed erroneous.

" As the conduct and practice of every person is governed, more or less, by the theory which he embraces, and as false theories have done irreparable injury to society in all ages, the opinion which I entertain of the dangerous tendency of the doctrine on which I have made the preceding remarks is the inducement for thus pointing out what appears to me to be some of its errors, and I hope will be considered as a sufficient apology for the freedom I have used; especially as there are but few things of more importance, or more to be desired in this world, than the establishment of truth on a subject which has the welfare of mankind for its object."

